# Deep Learning and Attention Mechanism-based Prediction of Vaginal Invasion in Early-Stage Cervical Cancer

**DOI:** 10.2174/0115734056392471251103125700

**Published:** 2025-11-28

**Authors:** Qing Xu, Chao He, Xinyang Zhu, Yuwei Xia, Feng Shi, Changyi Guo

**Affiliations:** 1 Department of Medical Techniques, Shaanxi University of Chinese Medicine, Shaanxi, Xianyang, 712046, China; 2 Xixian New Area Rimag Medical Diagnosis Center, Shaanxi, Xianyang, 712000, China; 3 Department of Radiology, The Second Affiliated Hospital of Shaanxi University of Chinese Medicine, Shaanxi, Xianyang, 712083, China; 4 Shanghai United Imaging Intelligence, Shanghai, 200030, China

**Keywords:** Cervical cancer, Radiomics, Deep learning, Radical trachelectomy, Magnetic resonance imaging, Colposcopy

## Abstract

**Introduction:**

This study introduces a novel fusion of 3D ResNet classification and Grad-CAM visualization to predict vaginal invasion in early-stage cervical cancer using T2WI-MRI, enhancing diagnostic accuracy while enabling anatomical localization of invasive lesions.

**Methods:**

This retrospective study analyzed sagittal T2WI from 160 patients with pathologically confirmed stage IB-IIA cervical cancer to predict vaginal invasion. Following an 8:2 training-test split, radiomic features were extracted from manually delineated intratumoral regions and four concentrically expanded peritumoral regions (1-4mm). Features selection by Pearson correlation and LASSO regression. Random forest models incorporating intratumoral and peritumoral (0-4mm) features were constructed, with ROC analysis identifying the optimal model. Subsequently, a 3D-ResNet architecture, enhanced with anisotropic convolutional layers and sophisticated data augmentation, was developed and optimized using the optimal ROI configuration. Model interpretability was facilitated using Grad-CAM, with performance assessed by AUC, sensitivity, specificity, accuracy, and precision.

**Results:**

The AIC-enhanced 3D ResNet-18 model, integrating intratumoral and 3mm peritumoral regions, showed superior test performance (AUC: 0.784, Sensitivity: 0.650, Specificity: 0.765, Accuracy: 0.611, Precision: 0.686) versus the baseline (AUC: 0.742), representing a 6% AUC improvement. Grad-CAM heatmaps identified diagnostically relevant regions within the tumor microenvironment, enhancing biological plausibility and model interpretability.

**Discussion:**

This attention-integrated 3D ResNet-18 framework (AUC=0.784) facilitates non-invasive vaginal invasion detection for fertility-sparing decisions, validated through Grad-CAM tumor localization; however, derivation from a single-center cohort warrants external validation and prospective studies before clinical translation.

**Conclusion:**

This preliminary study demonstrates promising deep learning performance (3D ResNet-18+Grad-CAM+AIC) for vaginal invasion assessment, despite moderate n; however, a single-center retrospective design limits generalizability.

## 1 INTRODUCTION

Cervical cancer is the fourth most common malignant tumor among women worldwide, posing a significant threat to women’s health [1]. Studies indicate that cervical cancer is the second leading cause of death among young women aged 20 to 39 [2]. Fertility preservation is a critical concern for patients with early-stage cervical cancer, particularly for young patients with fertility intentions. According to the International Federation of Gynecology and Obstetrics (FIGO 2018) guidelines [3], vaginal invasion is a key criterion for staging. The National Comprehensive Cancer Network (NCCN) recommends that patients in stage IB with a tumor size ≤ 2 cm, reproductive age < 45 years, no clinical evidence of impaired fertility, and no evidence of metastasis on imaging may opt for fertility-preserving treatments such as cervical resection [4]. Therefore, accurate staging is essential for determining appropriate surgical methods for fertility preservation in cervical cancer patients.

Colposcopy is a commonly used method for examining cervical abnormalities. It provides visualizations of the cervix through colposcopy images [5, 6], including saline, acetic acid, and iodine imaging. Although official institutions have released standards and quality control measures for vaginal colposcopy practice [7, 8], the accuracy of this practice largely depends on the subjective experience of clinicians, resulting in poor reproducibility [9]. Furthermore, visual inspection alone has been reported to be inaccurate, even for experienced clinicians [10, 11]. The gold standard for diagnosing cervical lesions is colposcopy-guided biopsy followed by pathological confirmation. However, this method is invasive and may lead to complications such as bleeding or infection. Thus, there is a need for a non-invasive, objective, and accurate cervical examination method. Magnetic resonance imaging (MRI) offers high soft-tissue resolution and avoids radiation exposure. It can demonstrate the extent of tumor invasion in the cervix and whether it has invaded the vagina or uterus through changes in magnetic resonance signals, providing a basis for clinical staging of cervical cancer. However, when lesions exhibit mild erosive, congestive, or inflammatory symptoms, the accuracy of MRI in evaluating upper vaginal invasion is limited [12, 13].

Radiomics, a rapidly evolving field, has gained recognition for analyzing medical images by extracting quantitative features [14]. However, the standard radiomics workflow requires retesting for stability, which can lead to variability and inconsistent reproducibility [15]. Prior studies predominantly focused on intratumoral radiomics, potentially neglecting critical biological information from the peritumoral microenvironment. Our study combined intratumoral and peritumoral regions to predict early cervical cancer vaginal invasion and provide more information related to tumor heterogeneity. Deep learning, as a state-of-the-art method, can automatically extract abstract and deep information from medical images, achieving high classification performance and providing objective and accurate diagnostic insights [16-19]. Based on colposcopy, deep learning models have achieved significantresultsinscreening, diagnosing, andevaluating postoperative efficacy in cervical cancer [20, 21]. However, the use of deep learning models to predict vaginal invasion in cervical cancer patients based on peritumoral regions in MRI images remains underexplored. Traditional CNN models act as “black boxes,” providing limited insight into the spatial or morphological features driving predictions (*e.g.*, tumor margin irregularity or stromal infiltration). This hinders clinical trust and utility. We integrate Gradient-weighted Class Activation Mapping (Grad-CAM) and attention heatmaps to visualize critical regions influencing predictions. This enhances clinical interpretability. Therefore, deep learning models integrating attention mechanisms that integrate intratumoral and peritumoral regions may improve the accuracy of predicting vaginal invasion in early-stage cervical cancer patients.

In this study, deep learning models integrating residual convolutional neural networks (ResNets) with MRI images of intratumoral and peritumoral regions were developed to evaluate vaginal invasion in early-stage cervical cancer patients. This approach aims to comprehensively reflect tumor heterogeneity, improve the diagnostic rate for vaginal invasion, and guide individualized treatment, such as radical trachelectomy, for patients seeking fertility preservation.

## 2 MATERIALS AND METHODS

This retrospective study was approved by the institutional review board, and informed consent was waived due to the retrospective nature of the study (Ethical approval number: LW2024011-1).

### 2.1 Study Population

This retrospective study enrolled patients with pathologically confirmed cervical cancer, radiologically staged as IB-IIA according to the FIGO 2018 staging system, at the Second Affiliated Hospital of Shaanxi University of Chinese Medicine between January 2019 and January 2025. In accordance with the 2019 updated clinical practice guidelines in oncology, cervical cancer cases were classified into three distinct phases: (1) early-stage disease (IA-IIA), (2) locally advanced disease (IIB-IVA), and (3) metastatic disease (IVB). The age of the patients is between 35 and 70 years.

Inclusion criteria were as follows: 1) Patients with pathologically confirmed early-stage cervical cancer (FIGO stages IB and IIA); 2) Availability of complete T2-weighted imaging (T2WI) images; 3) Patients who underwent radical hysterectomy and bilateral pelvic lymph node dissection. Exclusion criteria were as follows: 1) Patients with a history of other tumors; 2) Patients who received preoperative neoadjuvant chemotherapy or radiotherapy; 3) Poor MRI image quality or severe motion artifacts; 4) Tumors too small to outline the region of interest (ROI) in MRI images.

A total of 160 cervical cancer patients were retrospectively included from the Second Affiliated Hospital of Shaanxi University of Chinese Medicine. MRI images were retrieved from the hospital’s medical record system for subsequent analysis. The experimental flowchart of this study is illustrated in Fig. (**1**).

### 2.2 Grouping Criteria

Based on pathological examination findings, early-stage cervical cancer patients were stratified into two cohorts: the vaginal invasion-negative group (designated as 0) and the vaginal invasion-positive group (designated as 1).

### 2.3 Image Acquisition

The study utilized T2WI images. To avoid information loss, medical Digital Imaging and Communication (DICOM) images were directly acquired from Picture Archiving and Communication Systems (PACS) without compression or downsampling. Acquisition parameters were as follows: All patients were examined using a Siemens 3.0T Skyra MRI scanner with an 8-channel phased-array abdominal coil. Scanning parameters included sagittal T2-weighted imaging with a repetition time (TR)/echo time (TE) of 4700/48 ms, a field of view (FOV) of 320 × 320 mm, an excitation number (NEX) of 2, a slice thickness of 5 mm, and a slice spacing of 1 mm.

### 2.4 Deep Learning Analysis

#### 2.4.1 Image Segmentation

The uAI Research Portal (Version: 20231115, http://urp.united-imaging.com/ [22]) was used to manually segment the tumor layer by layer in sagittal T2WI images. Other sequences were used for areas with blurred boundaries. The ROI included bleeding, necrosis, or cystic areas within the tumor while avoiding normal muscle membranes near the tumor tissue. The ROI was used to remove cervical mucus based on a threshold. The study employed the pad function in the NumPy library (Version: 1.21.2, https://numpy.org/doc/ stable/reference/generated/numpy.pad.html) to expand the segmented original ROI at intervals of 1 mm (up to 4 mm) and manually erased parts that expanded into adjacent organs. An associate chief radiologist with 8 years of experience in gynecological pelvic MRI diagnosis performed the segmentation. Another associate chief radiologist with 10 years of experience verified the ROI segmentation results. Discrepancies in segmentation were resolved through consensus discussions between two radiologists to determine the final delineation. The segmentation process for intratumoral and 1–4 mm peritumoral regions is illustrated in Fig. (**2**).

#### 2.4.2 Radiomic Feature Extraction and Selection

1) Radiomic Feature Extraction

Following manual segmentation and morphological dilation of intratumoral and 1–4 mm peritumoral regions on sagittal T2-weighted imaging (T2WI) for all patients, radiomic features were extracted using 24 computational filters. A total of 2,264 features were quantified, encompassing four categories: first-order statistical features, shape-based features, texture features (derived from gray-level co-occurrence, run-length, and size-zone matrices), and wavelet transform features.

2) Radiomic Feature Selection

The selection process comprised two sequential steps:

(1) Univariate Correlation Analysis: Features were initially screened by calculating Pearson correlation coefficients with vaginal invasion status (binary outcome). Features demonstrating statistically significant associations (*p* < 0.05) were retained as candidate predictors.

(2) LASSO Regularization: A least absolute shrinkage and selection operator (LASSO) regression model with 5-fold cross-validation was implemented to optimize the regularization hyperparameter (**λ**). The optimal **λ** value was determined by minimizing the cross-validation error, thereby selecting features with non-zero coefficients that exhibited robust associations with vaginal invasion.

#### 2.4.3 Radiomic Model Construction

Using the selected features, five distinct predictive models were developed via a random forest classifier: 1) Intratumoral Model: Based solely on intratumoral features. 2) Four Peritumoral Models: Integrating intratumoral features with peritumoral features from progressively expanding regions (1 mm, 2 mm, 3 mm, and 4 mm beyond the tumor boundary). Model performance in predicting vaginal invasion was evaluated using receiver operating characteristic (ROC) analysis. The model achieving the highest area under the curve (AUC) was identified as the optimal predictive framework.

#### 2.4.4 Data Processing

During processing, grayscale values of the medical images were linearly normalized to the range [0, 1], a key step for improving model training efficiency. Images were resized to fit ResNet’s input specifications. Additionally, due to the layer thickness of MRI images (3–10 mm), the anisotropic convolutional (AIC) network mechanism was applied to sample the MR images at a spatial resolution of 0.688 × 0.688 × 5.5 mm. Data augmentation techniques, including translation, rotation, scaling, and flipping, were employed to address the limited number of training images.

#### 2.4.5 Training Process of the Deep Learning Classification Model

To optimize model performance on the training set, a binary classification model was developed using the 3D ResNet-18 network, which comprises an 18-layer deep residual network. Residual blocks, each containing two sets of 3 × 3 × 3 convolutional layers, batch normalization, and ReLU activation functions, were used to address deep network training challenges. These blocks are meticulously designed to learn complex spatial hierarchies from volumetric data, making the model highly suitable for tasks involving 3D data inputs. The ResNet-18 architecture offers distinct advantages through its residual learning framework:1) Enhanced Gradient Propagation: Skip connections enable direct addition of input signals to subsequent layer outputs. This structural design facilitates smoother gradient flow during backpropagation, effectively mitigating the vanishing gradient problem inherent to deep neural networks. 2) Optimized Training Dynamics: Accelerated Convergence: The residual learning mechanism simplifies network optimization, yielding faster convergence compared to non-residual architectures.Computational Efficiency: With its streamlined 18-layer structure (*vs*. deeper variants like ResNet-50/101), the model demonstrates reduced computational resource requirements. Under identical hardware configurations, ResNet-18 achieves faster training cycles, particularly advantageous in resource-constrained environ-ments. 3) Robust Feature Representation: Residual learning enhances the model's capacity to capture hierarchical feature representations. While effectively learning discriminative patterns from training data, it maintains strong generalization performance on test sets by prioritizing intrinsic data relationships over superficial training set memorization.4) Overfitting Mitigation:Compared to deeper ResNet iterations, ResNet-18 exhibits inherent architectural regularization. This balance between model complexity and parameter count reduces overfitting risks while preserving essential feature extraction capabilities.

Additionally, the architecture’s robustness and adaptability are enhanced by pooling and down-sampling operations, which can help to reduce dimensional complexity and computation without losing critical volumetric information. This is essential for achieving high accuracy in binary classification tasks.

During model training stage, initial features were extracted through convolutional and pooling layers. The model was then fine-tuned to adapt to a specific medical image dataset. The study used Adam optimizer to retrain the model and employed the cross-entropy loss function to guide backpropagation. The learning rate was set at 0.0001, with other parameters detailed in Table **1**. Early stopping criteria were applied to prevent overfitting, terminating training when no further improvement in loss or accuracy was observed. The model with the lowest test loss was selected, allowing up to 100 iterations.

#### 2.4.6 Model Visualization Algorithm

To improve the interpretability of the model, Gradient-weighted Class Activation Mapping (Grad-CAM) was embedded as an attention mechanism. Once the model produces classification results, features from the final convolutional layer were extracted and calculated by deriving the gradient of the target class score relative to the feature map of the last convolutional layer. These gradients were globally averaged and merged to generate weights reflecting the importance of each feature map, which were then aggregated to produce a heatmap. The heatmap was overlaid on the original input image, highlighting regions of interest for the model and providing intuitive visualization. Based on the lesion area of clinical concern, the interpretability of the model was improved, and its potential value in diagnosing vaginal invasion by cervical cancer was demonstrated. The network structure diagram is shown in Fig. (**3**).

### 2.5 Statistical Analyses

The sample size was calculated using the AUC difference method with the following parameters: a significance level (α) of 0.05, a type I error probability (β) of 0.2, an expected model performance (AUC_1_) of 0.8 based on prior literature, a clinically meaningful null hypothesis (AUC_0_) of 0.65, and an 8:2 ratio for the training-to-test set split. The calculation yielded a minimum required sample size of 113.

All statistical analyses in this study were performed by uAI Research Portal (Version: 20231115; accessible at http://urp.united-imaging.com/). Radiomic features were initially screened by calculating Pearson correlation coefficients with the binary outcome of vaginal invasion status. Features demonstrating statistically significant associations (*p* < 0.05) under a prespecified significance threshold (**α**= 0.05) were retained as candidate predictors. Subsequently, a least absolute shrinkage and selection operator (LASSO) regression model was implemented, employing 5-fold cross-validation to optimize the regularization hyperparameter (**λ**). The optimal **λ** value was determined by minimizing the cross-validated mean squared error (MSE), thereby selecting features with non-zero coefficients that exhibited robust associations with vaginal invasion status. Model performance was evaluated using the area under the receiver operating characteristic curve (AUC), accuracy, sensitivity, specificity, and precision.

## 3 RESUITS

### 3.1 Comparison of Classification Performance for Different Peritumoral Regions

Based on different peritumoral radiomics models, the classification performance for predicting vaginal invasion is shown in Table **2**. Finally, 5~10 features are retained from different peritumoral regions. From the 3 mm peritumoral region, five radiomics features were extracted, including First-order minimum of curvature flow-filtered images (Curvature Flow_firstorder_Minimum), First-order minimum of discrete Gaussian-filtered images (DiscreteGaussian_firstorder_Mini- mum), Least axis length from original image morphology (Original_shape_LeastAxisLength), Gray-level non-uniformity in box sigma-filtered images (BoxSigmaImage_glszm_ GrayLevelNonUniformity), Normalized size-zone non-uniformity in 3D log-sigma-0.5-mm transformed images (LogSigma05mm3D_glszm_SizeZoneNonUniformityNormalized). The radiomics model constructed from these features demonstrated superior performance in both the training and testing sets (AUC: 0.939 for the training set and 0.845 for the testing set). The results are shown in Table **2**.

### 3.2 Performance Evaluation of Neural Network Models

A 3D ResNet-18 model was developed by integrating radiomic features from both intratumoral regions and 3 mm peritumoral zones on T2-weighted imaging (T2WI) to predict vaginal invasion status in cervical cancer. The model demonstrated promising predictive performance, achieving an AUC of 0.742 (73.1% sensitivity, 60.9% specificity), thereby preliminarily validating its clinical utility and establishing a foundation for further investigations.

Prior to deep learning analysis, adaptive image contrast (AIC) preprocessing was applied, which enhanced model efficacy by 6%, underscoring the necessity of image preprocessing, particularly in scenarios with limited sample sizes. Detailed quantitative results are presented in Table **3**. The receiver operating characteristic (ROC) curves for testing set are shown in Fig. (**4**).

### 3.3 Classification Performance of Using Grad-CAM

The Grad-CAM visualization results are depicted in Fig. (**5**), demonstrate the heatmap overlaid on the original imaging data, highlighting the critical anatomical regions prioritized by the model during vaginal invasion status prediction in cervical cancer. Notably, these high-attention regions (indicated by red and yellow hues) exhibit spatial concordance with tumor boundaries, while areas of lower predictive contribution are represented in blue.

## 4 DISCUSSION

In this study, a 3D ResNet-18 model was established to distinguish whether the vagina was invaded in patients with early-stage cervical cancer. The results showed that the deep learning model, based on MRI images and attention mechanisms, demonstrated good performance in correctly identifying vaginal invasion in these patients, achieving an AUC of 0.784, which has important implications for preserving patients’ fertility.

Radiomics is a non-invasive method for the clinical diagnosis of benign and malignant tumors and their staging [23, 24]. Radiomics has become a hot topic in recent years. Recent studies have reported that the peritumoral region also provides rich information on tumor heterogeneity [25, 26]. Researcher found that radiological structural analysis based on pre-processed dynamic enhanced MRI of the tumor and the peritumoral region successfully predicted pathologically complete responses to neoadjuvant chemotherapy [27]. This study confirmed that the radiomic model combining the tumor and the peritumoral region is superior to the single-region model, thus improving prediction accuracy and increasing the stability of the model. The study showed that radiomic features from within 3 mm of the tumor perimeter had the best predictive performance. This is consistent with the findings of Shi [28]. From a pathological perspective, tumor cells tend to migrate from the primary region to the peritumoral region, resulting in changes in lesion morphology on MRI images.

In recent years, deep learning technology has made significant progress in the field of medical image analysis, especially in the early diagnosis of neurological diseases and tumors. These studies not only improve the diagnostic performance of the model, but also enhance the clinical practicability through the transparent decision-making process, which provides an important reference for the development of medical AI in the future.For example, Dual-3DM3AD [29] combines Transformer and 3D convolutional neural network (CNN) for multi-categorical early diagnosis of Alzheimer's disease, and uses Transformer's long-range dependent modeling ability to capture the global features of brain structural changes, while extracting local details through CNN. Similarly, D2PAM [30] uses Dual Patch Attention to analyze pre-seizure electroencephalogram or functional magnetic resonance signals, and improves prediction sensitivity through spatial-temporal dual-path modeling. XAI-RACapsNet [31] introduces Capsule Network and O-net ROI segmentation to achieve accurate localization and classification of lesion areas in breast cancer detection. These architectural designs reflect the current trend in medical AI research: hybrid models to compensate for the limitations of a single network, thereby improving generalization capabilities in small samples or complex data environments.

The ResNet model has been widely used in model training because of its excellent image classification performance. ResNet introduces skip connections, which address the issues of gradient disappearance and training difficulties in deep neural networks, allowing efficient training of deeper networks [32]. On the other hand, visualization is particularly important for deep learning in the medical field, as it can help researchers understand the features encoded within these networks. In this study, the ResNet model combined AIC and Grad-CAM technology (used to highlight regions of interest while suppressing background areas) to train the network. The heatmap can assess the location of features associated with cervical cancer, with lesions exhibiting high intensity on the heatmap, thereby making the lesion areas more prominent. By generating this heatmap image, Grad-CAM technology can elucidate the internal logic of the ResNet model and its feasibility in medical imaging analysis. Zhang *et al*. [33] investigated the clinical utility of three heatmap generation techniques—Class Activation Mapping (CAM), Gradient-weighted CAM (Grad-CAM) and Grad-CAM++ in classifying relapsing-remitting multiple sclerosis, secondary progressive multiple sclerosis, and healthy controls using convolutional neural networks (CNNs). Their results demonstrated that Grad-CAM exhibited superior localization capability compared to CAM and Grad-CAM++, suggesting that integrating Grad-CAM with heatmap quantification methods and CNN models may represent a critical strategy for identifying disease progression in multiple sclerosis. In the present study, Grad-CAM-generated heatmaps revealed spatial overlap with tumor regions, further validating its robust localization performance. Chen *et al*. [34] developed a deep learning algorithm for detecting architectural distortions in breast imaging. Their approach employed Grad-CAM to automatically localize abnormalities and generate regions of interest (ROI), followed by a radiomics model for classification, with ROC curves constructed to evaluate diagnostic efficacy. The results indicated that heatmap-derived ROI achieved diagnostic accuracy comparable to manual ROI but were primarily applicable for binary detection of lesion presence.

Grad-CAM heatmaps into Clinical application scena-rios: 1) Initial screening assistance: Before the radiologist reads the images, the system automatically marks the areas with high response to Grad-CAM (such as the suspicious areas of tumor invasion) to shorten the diagnosis time. 2) Review of controversial cases: When physicians are not confident in the diagnosis (eg, FIGO stage IB micro-infiltration), heat maps are retrieved to verify whether the area of interest of the model is consistent with clinical suspicion.3) Teaching tools: Visualize the decision-making basis of the model to help residents understand the imaging characteristics of cervical cancer.

Deep learning-based medical image analysis has made significant progress in the diagnosis of neurological diseases. the Tri-M2MT [35] study improved the diagnostic accuracy of neonatal acute bilirubin encephalopathy through the fusion of multimodal MRI data, while Lao study [36] evaluated the adaptability of different models to the classification task of brain tumors and Alzheimer's disease from the perspective of data complexity, which together promoted the development of the field of intelligent diagnosis of medical imaging. At the same time, due to the limitations in classification performance of two-dimensional deep learning models, as a significant amount of spatial information from adjacent tumor slices can be lost [37], the learning approach using three-dimensional convolutional kernels in the convolutional neural network employed in this study offers distinct advantages.

On T2WI images, the cervix clearly displays the normal four-layer structure with four distinct signal layers: the cervical mucus (innermost layer) shows a high signal; the mucosal layer of the cervix shows a high signal (lower than that of the mucus); the cervical fibrous stroma (the connective tissue layer) exhibits a low signal; and the cervical muscle layer presents a moderate signal. When diagnosed as negative for vaginal invasion, the imaging shows T2WI isointensity with interruption in the adjacent fibrous stroma band. When diagnosed as vaginal invasion, the imaging shows T2WI isointensity with localized discontinuity in the adjacent fibrous stroma band, and the lesion involves the upper segment of the adjacent vagina. It is noteworthy that sagittal T2WI serves as the most clinically significant sequence for tumor characterization and staging, particularly in evaluating the extent of vaginal invasion by lesions. Consequently, the T2WI sequence was selected for inclusion in this study to ensure comprehensive pathological assessment and clinical relevance.

However, our research also has certain limitations. First, the number of subjects is limited. The study plans to include more data to further validate the predictive efficacy and stability of the model in future studies. Second, due to the limited availability of preoperative MRI enhanced images and DWI sequences, this study only analyzed plain scan images, which may underestimate the clinical value of deep learning. Third, owing to the inherent limitations of the retrospective study design, standardization of slice thickness parameters in original imaging data acquisition was unattainable. Partial volume effects arising from heterogeneous scanning equipment may compromise the precision of quantitative lesion feature analysis. To address these limitations, first, future studies will expand sample sizes and incorporate dynamic contrast-enhanced and multimodal fused images to refine radiomic analyses, enhance model predictive performance and robustness, and ultimately improve clinical applicability and objectivity of research findings.Second, future efforts will prioritize standardization across all workflow stages. This includes harmonizing protocols for image acquisition, preprocessing, ROI segmentation, feature extraction, and selection, as well as establishing unified evaluation criteria and metrics to ensure methodological consistency and comparability.Third, to further advance the comprehensiveness and accuracy of this research, we plan to implement machine learning-based fully automated segmentation of cervical tumors. This approach aims to minimize manual intervention, thereby improving segmentation precision, strengthening scientific rigor, and maximizing the practical utility of study outcomes in clinical settings.

## CONCLUSION

In summary, the 3D ResNet-18 + AIC + Grad-CAM model can effectively predict the preoperative prognosis of vaginal invasion in early-stage cervical cancer, which is helpful for radiologists to improve the accuracy of cervical cancer staging diagnosis and helps patients select more precise and individualized treatment.

## Figures and Tables

**Fig. (1):**
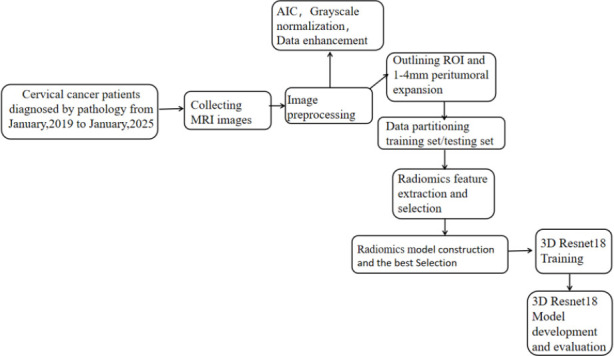
Flowchart of the experiment.

**Fig. (2):**
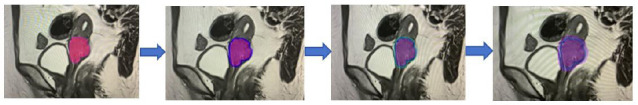
Example of the expansion process of peritumoral regions. Colorful rings indicate peritumoral regions, with each ring representing a 1 mm dilation.

**Fig. (3):**
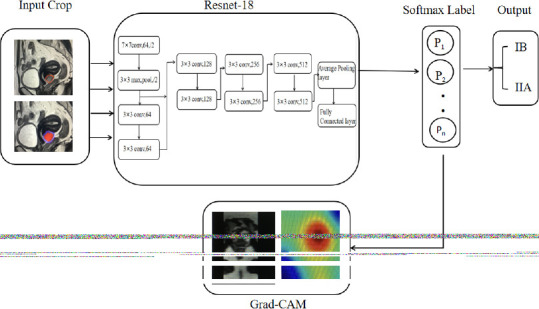
Diagram of the 3D ResNet-18 structure. The Grad-CAM mechanism generated attention maps during training. “conv” refers to the convolutional layer in the 3D ResNet-18. “Average Pooling” refers to the global average pooling layer. “Fully Connected Layer” refers to the fully connected layer. “SoftMax Label” represents the prediction probability of a certain type calculated by the SoftMax function. “Grad-CAM” refers to the differences between network attention regions and tumor regions.

**Fig. (4):**
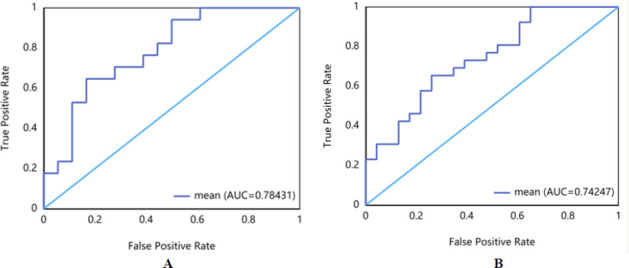
ROC curve of the 3D Resnet 18. **A: **AIC preprocessed images, **B: **Image without AIC preprocessing.

**Fig. (5):**
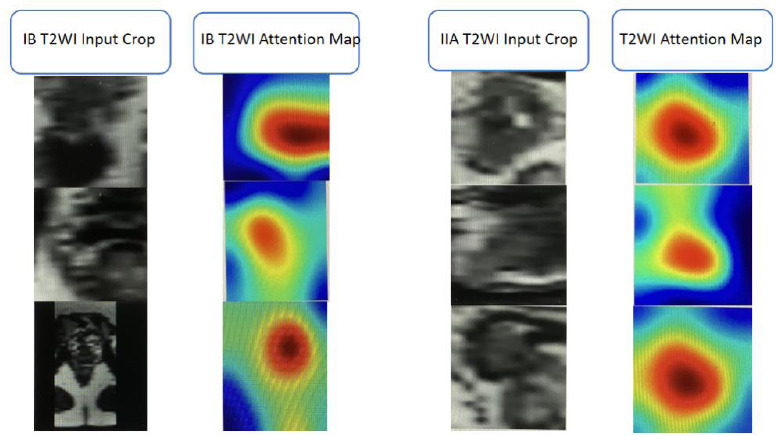
Examples of heatmap generation.

**Table 1:** Parameter setting.

**Hyperparameters**	**Value**
Optimizer	Adam
β1, β2	0.9, 0.999
Eps	1e-8
Weight decay	0.01
Learning rate	1e-4
Batch Size	64
Epoch	100

**Abbreviations:** Eps: Epsilon, β1:Exponential decay rate for the first moment estimates, β2:Exponential decay rate for the second moment estimates.

**Table 2:** Comparison of performance in distinguishing IB and IIA stage cervical cancer based on different peritumoral radiomics models.

**Expansion distance**	**Number of features**	**Set**	**AUC**	**95%CI**	**SEN(%)**	**SPE(%)**	**ACC(%)**	**PRE(%)**
0 mm	6	Training set	0.933	(0.892-0.973)	88.8	86.3	87.6	88.8
		Testing set	0.872	(0.751-0.931)	90.0	68.8	80.6	78.3
1 mm	5	Training set	0.874	(0.823-0.923)	73.8	80.0	76.9	78.7
		Testing set	0.854	(0.733-0.975)	80.0	78.9	79.5	80.0
2 mm	10	Training set	0.893	(0.846-0.946)	73.8	91.2	82.5	89.4
		Testing set	0.854	(0.727-0.981)	70.0	84.2	76.9	82.4
3 mm	5	Training set	0.939	(0.905-0.973)	90.0	77.5	83.8	80.0
		Testing set	0.845	(0.712-0.977)	87.0	68.4	79.5	75.0
4 mm	8	Training set	0.867	(0.813-0.921)	68.8	83.8	76.2	80.9
		Testing set	0.807	(0.652-0.961)	75.0	78.9	76.9	78.9

**Abbreviations:** AUC: area under the receiver operating characteristic curve; SEN: sensitivity; SPE: specificity; ACC: accuracy; PRE: precision.

**Table 3:** Performance comparison of ResNet-18 for predicting vaginal invasion status in cervical cancer (Testing set).

**DL model**	**AUC**	**95%CI**	**SEN(%)**	**SPE(%)**	**ACC(%)**	**PRE(%)**
3D ResNet-18 + Grad-CAM	0.742	0.640-0.843	73.1	60.9	67.3	67.9
3D ResNet-18 + Grad-CAM+ AIC	0.784	0.691-0.877	65.0	76.5	61.1	68.6

**Abbreviations:** ResNet: residual network; AIC: anisotropic convolutional; CAM: class activation mapping; AUC: area under the receiver operating characteristic curve; ACC: accuracy; SEN: sensitivity; SPE: specificity; PRE: precision.

## Data Availability

All data generated or analyzed during this study are included in this published article.

## LIST OF ABBREVIATIONS

FIGO: International Federation of Gynecology and Obstetrics
MRI: Magnetic Resonance Imaging
NCCN: The National Comprehensive Cancer Network
CNN: Neural Convolutional Network
ResNet: Residual Neural Network
Grad-CAM: Gradient Weighted Class Activation Mapping
LASSO: Least Absolute Shrinkage and Selection Operator
ROC: Receiver Operating Characteristic
DCA: Decision Curve Analysis
AUC: Area Under the Curve
HPV: Human Papillomavirus
T2WI: T2-weighted imaging
DWI: Diffusion Weighted Imaging
DICOM: Digital Imaging and Communication images
PACS: Picture Archiving and Communication Systems
TR: Repetition time
TE: Echo time
FOV: Field of view
NEX: Excitation number
SCCA: Squamous cell Carcinoma Antigen
WBC: White Blood Cell
RBC: Red Blood Cell
PLT: Platelet
Rad score: Radiomics score
SEN: Sensitivity
SPE: Specificity
ACC: Accuracy
PRE: Precision
AIC: Anisotropic Convolutional

## STANDARDS OF REPORTING

STROBE guidelines were followed
